# Mothers with Intellectual/Developmental Disabilities and Behavioral Health Conditions and Community Experts Provide Recommendations for Treatment/Services, Systems, and Research

**DOI:** 10.3390/ijerph20105876

**Published:** 2023-05-19

**Authors:** Joanne Nicholson, Shayna Mazel, Kristen Faughnan, Allie Silverman

**Affiliations:** 1 Institute for Behavioral Health, The Heller School for Social Policy and Management, Brandeis University, Waltham, MA 02453, USA; 2 Schneider Institutes for Health Policy and Research, The Heller School for Social Policy and Management, Brandeis University, Waltham, MA 02453, USA

**Keywords:** parenting, intellectual and developmental disabilities, behavioral health, treatment, health care, community engagement

## Abstract

Mothers with intellectual and developmental disabilities (IDD) are vulnerable to perinatal complications and adverse outcomes. Their vulnerabilities may also be exacerbated by co-occurring behavioral health (BH) conditions. Their wellbeing may be compromised by a lack of tailored treatments or by treatments and services that are inaccessible, irrelevant, and/or ineffective. A five-session virtual Ideas Lab workshop series was implemented to bring together diverse community experts (*n* = 30), including mothers with IDD/BH, to discuss the experiences of mothers and set priorities for treatment/services, systems, and research. Participants completed background and evaluation surveys and brainstormed, grouped, and ranked items of importance, which fell into two broad categories: (1) cross-cutting themes drawn from lived experience with recommendations applicable to all substantive domains (i.e., accessibility, diversity, adverse experiences and trauma, and trust) and (2) substantive themes with specific recommendations for treatment/services and systems (i.e., services and supports, peer support, provider practices and training, and systems navigation/transformation). Research recommendations were generated in all discussions and emerged in relation to all themes, underscoring the importance of including mother-driven questions and priorities in research agendas and strengthening researcher training and skills to engage mothers with IDD/BH and other community members actively, and in meaningful ways.

## 1. Introduction

Mothers with intellectual and developmental disabilities (IDD) are a “hidden” population [1] who are vulnerable to perinatal complications and adverse birth outcomes [2,3,4,5]. Intellectual and developmental disabilities are defined by the U.S. National Institutes of Health as chronic disabilities that originate at birth or in the developmental period with the ability to impair physical, intellectual, and/or emotional development [6]. The vulnerabilities of mothers with IDD may be exacerbated by co-occurring behavioral health (BH) conditions (e.g., anxiety, depression, psychotic disorders, and/or substance use) [7,8,9]. Their wellbeing and the wellbeing of their children are compromised by a lack of tailored treatments. Mothers with IDD/BH are, in most instances, not directly involved in providing their perspectives on their experiences or treatment needs in research [10]. Consequently, existing treatment, services, and supports may be inaccessible, irrelevant, and/or ineffective.

The goal of this project was to engage diverse community stakeholders (i.e., mothers, family members, advocates, service providers of various types, policymakers, and researchers) in exploring the experiences and needs of mothers with IDD/BH, making recommendations to address unmet needs and setting priorities for treatment/services, systems, and research. While motherhood and pregnancy do not always go hand-in-hand, the project focused primarily on the experiences of women who defined themselves as mothers with IDD/BH who had given birth to their children. Our ultimate intention was to build capacity for engaging mothers with IDD/BH and other stakeholders, all defined as “community experts,” in the development and testing of treatment and supportive service options that reflect their needs and preferences and that take into consideration their strengths and impairments as well as the immense stigma attached to mental illness and substance use.

The Maternal Mental Health Research Collaborative (MMHRC), founded in 2017, sits at the intersection of behavioral health (i.e., mental health and/or substance use disorders), intellectual and developmental disabilities (e.g., autism, ADHD, dyslexia, and learning disabilities), and maternal morbidity and mortality. The primary objective of the MMHRC is to build the capacity of mothers and other community experts to collaborate in patient-centered comparative effectiveness research, the dissemination of research findings, and informed health decision making. The MMHRC embraces an Experience-Based Co-Design (EBCD) participatory research approach, bringing mothers, community stakeholders, and researchers together to reflect on their experiences, identify priorities, and devise and test innovations in care [11,12,13].

### 1.1. Background

#### 1.1.1. Fertility Rates and Perinatal Complications

Women with IDD are bearing children at increasing rates [2,14,15], though their general fertility rates are slightly lower than those for women without IDD, depending on age [16]. The age-specific fertility rate of 18- to 24-year-old women with intellectual and developmental disabilities has been found to be similar to that of women without IDD [14]. Women with IDD are at increased risk for perinatal complications and adverse birth outcomes, such as Caesarean deliveries, early labor, preterm births, stillbirth, low birth weight, and lower Apgar scores [2,3,14]. Among women with singleton live births, those with IDD are younger, with higher rates of poverty, mental health issues, and medication use during pregnancy [14]. Non-Hispanic Black and Hispanic women with IDD are significantly more likely to have stillbirths and much higher labor and delivery charges than other women [3,17].

#### 1.1.2. Co-Occurring Disorders

Mental health and substance-related disorders are found at substantially higher rates among adults with IDD [8,9,18,19]. In individuals with both IDD and substance-related disorders, the rate of psychiatric comorbidity is very high, with the most common conditions being anxiety disorders (over two-thirds), affective disorders (nearly half), psychotic disorders (over one-third), and personality disorders (nearly one-quarter of those with both IDD and substance-related disorders) [8]. Perinatal mental disorders contribute to maternal morbidity and mortality globally and are associated with poor infant outcomes [20].

#### 1.1.3. Treatment and Service Disparities

Mothers with disabilities also face considerable disparities in treatment and service systems [21]. Healthcare providers may not be prepared to meet the needs of mothers with IDD in preventive care and routine assessments [22]. Empirically based treatments focusing on the reproductive health of this group of women may not be available [14]. Recommendations have been made, for example, for further attention to increasing the recognition of mental health conditions and substance-related disorders among individuals with IDD to improve treatment access and outcomes [8,9,23]. Effective, evidence-based treatments for perinatal mental health (e.g., interpersonal psychotherapy, cognitive behavioral therapy) are likely not tailored to meet the needs and preferences of mothers or pregnant women with behavioral health conditions who are living with IDD [24]. The management of medications that are helpful for mothers receiving mental health treatment may be challenging for those with IDD [25].

#### 1.1.4. Lack of Support

In addition, women with IDD lack accessible informational support, emotional support, and social companionship during the perinatal period [15,26]. Factors influencing informational support include information format, the individual’s level of autonomy, and caregiver involvement [15]. Decisions about health and health care are often made in the context of relationships with family members, friends, and providers. Available social support is not always perceived as beneficial, however, if the sources of support or information have negative attitudes and beliefs about mothers with IDD [15,26]. Additional barriers to treatment are related to low health literacy, poor knowledge of the health care system, language, and culture [20].

### 1.2. Objective

A five-session virtual Ideas Lab workshop series was implemented to bring diverse community experts, including mothers, together to discuss the issues facing mothers with IDD/BH in facilitated sessions specifically designed to support the engagement of participants with IDD and behavioral health conditions. The primary objective was to develop an actionable agenda with the next steps in treatment/services, systems, and research, informed by community experts. The purpose of this article is to describe the Ideas Lab process for engaging mothers with IDD/BH and other community experts and to outline the recommendations for future developments that emerged in Ideas Lab workshop sessions.

## 2. Materials and Methods

The Ideas Lab workshops were hosted by the Maternal Mental Health Research Collaborative (MMHRC) at Brandeis University and funded by the U.S. Patient-Centered Outcomes Research Institute (PCORI). Given the COVID-19 pandemic, the project was developed intentionally to take advantage of virtual engagement options. In fact, face-to-face sessions during this time period were restricted due to the pandemic. Virtual sessions allowed us to support the participation of diverse people—diverse both in terms of geography as well as in accommodations (e.g., closed captioning, text interactions in the chat, recording for replay by participants who wished for recordings). We employed qualitative methods in this study that were appropriate for this exploratory work [27]. The Brandeis University IRB reviewed proposed procedures and determined the project to be a community engagement initiative, rather than human subject research; consequently, participants were well-informed about the project, but written consent was not required or obtained. Participants were offered a gift card or honorarium equal to USD 50 per hour of time engaged. Those who were mothers with children at home received a gift box of age-appropriate toys or activities to be used while mothers were engaged in virtual sessions.

### 2.1. The Implementation Team

The Implementation Team was comprised of diverse stakeholders who prepared for Ideas Lab sessions and anticipated and implemented strategies for encouraging and supporting the virtual participation of individuals with disabilities, specifically IDD and behavioral health conditions. The Ideas Lab Implementation Team included six members who represented the state disability services agency, a community agency serving mothers, a national advocacy organization, and the research team, comprised of senior investigators and trainees. It is important to note that participants in the Implementation Team, as well as participants in the Ideas Lab workshop sessions, reflected multiple perspectives. The majority were mothers themselves and/or family members of individuals with disabilities; the group included a policymaker, service providers with diverse training and expertise, and health policy and services researchers. The Implementation Team met regularly for three months to specify the purpose and structure of the Ideas Lab workshops, think strategically about whom to invite and how to encourage and support their participation and develop potential procedures and topics for discussion. The goals identified by the Implementation Team included building communities, sharing experiences, identifying service and research needs, developing potential collaborations, and developing strategies for engaging mothers with IDD/BH in research. Following these initial conversations, the Implementation Team then met regularly for two months with team members from Knowinnovation, Inc., (KI) who were contracted to facilitate the Ideas Lab workshop sessions. The rationale for having external facilitators was twofold. First, the KI team members had extensive experience and expertise in facilitating diverse virtual meetings for many different groups and organizations; the Implementation Team was interested in learning from their experience. Second, having external facilitators allowed Implementation Team members the freedom to participate fully in Ideas Lab sessions and pay greater attention to the contributions of other attendees. Implementation Team members partnered actively with KI facilitators to set the agenda for sessions and support the engagement of all attendees.

### 2.2. Recruiting Participants and Supporting Participation

The Implementation Team developed a process for inviting a snowball, convenience sample of Ideas Lab participants. The goal was to include up to 30 individuals, split as evenly as possible among mothers with IDD/BH, family members, advocates, providers, policymakers, and researchers (i.e., experienced and junior investigators/trainees). Potential participants were solicited via the Implementation Team’s extensive local, regional, and national networks, with consideration paid to diversity and inclusivity. Invitations were sent in late January 2022 and included information about the timing and purpose of the workshop sessions, as well as incentives to contribute time and effort to the project. Mothers, advocates, providers, policymakers, and researchers were asked to respond by briefly describing themselves, their interests, and what they hoped to gain by attending. As mentioned previously, many invitees were known to reflect multiple identities (e.g., advocates, providers, or researchers who were also mothers or family members). In addition, mothers were contacted by telephone by a member of the Implementation Team who conducted one-on-one preparation calls with them. These conversations included opportunities to make a personal connection with a team member, describe the purpose and structure of the Ideas Lab workshops, discuss expectations and accommodations that might be helpful to support participation, consider logistics (e.g., preparation of a brief bio paragraph and choice of gift card), and answer any questions. Participants submitted brief written biographical statements that were distributed in advance of the first Ideas Lab session.

### 2.3. The Ideas Lab Workshop Sessions

Working together with the KI facilitators, the Implementation Team outlined the structure for a series of five two-hour online sessions using the Zoom videoconferencing platform, which took place every Friday afternoon for five weeks in March and April 2022. Drawing from the initial Implementation Team goals, sessions focused on (1) participants getting to know each other and discussing expectations for the workshop series; (2) sharing stories about challenges faced and what would have helped; (3) identifying unmet needs; (4) brainstorming ways to meet those needs, including identifying existing resources and those that could be adapted or developed; (5) specifying relevant positive outcomes for mothers and their families; and (6) creating action plans and next steps for further collaboration. In each session, an agenda or plan for the session was provided, along with a recap of the prior session in the second through fifth sessions. In the first workshop session, following an initial full-group convening, participants were placed in one of five breakout rooms with others in the same initial role designation (e.g., mothers, providers, etc.) to facilitate introductions and support relationship building. In subsequent workshop sessions, following the initial full-group convening, participants were placed in one of five breakout rooms, in prearranged groups of five or six diverse participants (e.g., to ensure a mix of stakeholders) or as selected by participants interested in particular topics. Full group sessions were facilitated by the KI facilitators and Implementation Team members. Breakout sessions were facilitated by a diverse group of volunteers (i.e., mothers, providers, and researchers). Facilitators were prepared by the Implementation Team in advance and were alerted to opportunities to encourage and support the participation of all attendees in breakout sessions. Power dynamics that could have been at play, given the various roles and positions of participants, were attended to in several ways. Accommodations were made for all participants to support communication and give participants “a voice”. The pool of facilitators included participants representing all roles (e.g., mothers, providers, researchers). A scribe was assigned to each breakout room, with this role filled by community experts as well as project staff.

The Implementation Team met between workshop sessions to debrief from the prior session and confirm plans for the next session. The Implementation Team also sent emails to participants after each session with information about preparing for the next session, office hour opportunities during the coming week, and other miscellaneous information deemed relevant (e.g., resources as identified by participants). Before each session, the Implementation Team emailed participants an agenda, a reminder of questions to consider ahead of the session, and the Zoom link. Implementation Team members also communicated with individual participants between sessions if they had questions or something they wanted to discuss.

### 2.4. Data Collection

#### 2.4.1. Preliminary Survey and Participation

In confirming their participation in the Ideas Lab workshops, stakeholders completed a brief preliminary survey, providing their personal identification (i.e., checking all categories that applied including mother, advocate, provider, policymaker, and researcher), organizational affiliation (if relevant), and a brief note about their background and interest in the topic of IDD and behavioral health for mothers. Participation was tracked over the course of the Ideas Lab workshop sessions.

#### 2.4.2. Detailed Workshop Notes

Detailed notes were written by designated scribes in each of the five workshop sessions in both large group and breakout sessions using a prepared template. Notes were reviewed by the project and Implementation Team members between sessions to inform preparation for subsequent sessions and in follow-up debriefing sessions after the Ideas Lab workshops had ended.

#### 2.4.3. Brainstormed Statements

During workshop sessions, participants generated ideas (i.e., brainstormed statements) in response to session topics and planned probes related to challenges faced, what would or did help, unmet needs, resources to address those needs, and what “success” would look like. Participants were asked to identify and group emerging themes in an iterative process, with prior sessions informing subsequent sessions. Participants then identified the “most important” items among these through a rating process using a virtual whiteboard.

#### 2.4.4. Post Ideas Lab Survey

Following the completion of the Ideas Lab workshops, participants completed an online evaluation survey. Ten items from the Quality of Patient-Centered Outcomes Research Partnerships Instrument were adapted for use in evaluating engagement in planning sessions and Ideas Lab sessions [28]. These Likert items (1 = “not at all” to 3 = “very much”) related to participants’ understanding of the purpose of the workshops, nature and quality of their participation, comfort, ability to communicate with and relate to other participants, and overall impact of participation. Additional survey items included recommendations for improvement (i.e., “Please suggest at least one way workshops could be improved in the future.”) and continued engagement with the group (e.g., a checklist including email, list serve, social media, research participation, etc.).

### 2.5. Analysis

Preliminary survey data regarding background characteristics and interests were analyzed quantitatively to provide descriptions of Ideas Lab participants. Responses to items in the follow-up survey were analyzed quantitatively, where appropriate, with responses to open-ended items regarding recommendations for improvements systematically reviewed by the Implementation Team and project team.

The project team employed a phenomenological approach to the analysis of Ideas Lab workshop data [27]. Templates with detailed workshop session notes taken by designated scribes were systematically reviewed to identify major organizing ideas and summarized by project team members between Ideas Lab sessions to provide participants with a synopsis of prior sessions and lay the groundwork for further sessions. Statements generated by participants freely in workshop sessions, guided by the framework of workshop session topics (e.g., identifying challenges, unmet needs, etc.), were grouped by themes and compiled over time in an iterative process by the project team. Redundancies were removed. The statements were then provided to workshop participants using a virtual whiteboard. Participants applied colored dots to those statements considered most important. Thematic groupings and relative importance were reviewed by project team members by hand and summary memos were written for review by the Implementation Team. Summaries were provided to Ideas Lab participants for final reviews and feedback. The trustworthiness of findings was established, therefore, through collaboration with participants, the preparation of memos with rich descriptions, peer review, and member checking.

It is important to note that, because participants provided their perspectives reflecting multiple roles (e.g., mother, advocate, provider), statements were oftentimes attributed in the analysis to “participants” in general, rather than characterized as being offered by “a mother” or “a provider,” unless the participant clearly specified the perspective from which they were speaking.

## 3. Results

### 3.1. Participants

An average of 26 or 27 participants of the total 30 invitees attended each of the five Ideas Lab workshop sessions, along with 6 members of the Implementation Team and project staff. Participants may have missed particular sessions because of schedule conflicts; these absences were random. The majority of participants identified as women (*n* = 29 of 30), reflecting the focus on mothers with IDD and behavioral health conditions and the preponderance of women in human services provider and policymaker roles. In specifying primary roles/identification, 6 indicated their primary identification as “mother”, 9 listed “provider”, and 15 indicated “researcher”. However, multiple roles were frequent. A total of 5 of the 6 mothers described themselves as advocates and 2 were peer specialists. A total of 5 of the 9 service providers indicated that they were advocates, while 4 were mothers and 3 were policymakers. Of the 15 researchers, 10 were also mothers, 3 were providers, 2 served as advocates, and 1 also functioned as a policymaker. In providing reasons for wanting to participate in the Ideas Lab workshops, many expressed an interest in engaging with diverse stakeholders to build opportunities to connect and collaborate; many also wanted to improve care and address inequities for mothers with IDD and behavioral health conditions. Participants were eager to share both personal and professional knowledge and experience. Four participants reported having children or family members with disabilities as well.

### 3.2. Emerging Themes

Themes and recommendations were informed, reviewed, and validated by Ideas Lab participants representing diverse and, at times, multiple perspectives. The group generated 148 brainstormed statements that fell into two broad categories: (1) cross-cutting themes drawn from lived experience, with recommendations applicable to all substantive domains (i.e., accessibility, diversity, adverse experiences and trauma, and trust) and reflecting “how” things are done and (2) substantive themes with specific recommendations for treatment/services and systems (i.e., services and supports, peer support, provider practices and training, and systems navigation/transformation), reflecting “what” things are done or could be done. It is important to note that these categories were not mutually exclusive. For example, accessibility issues were noted in discussions regarding interventions. Research recommendations were generated in all discussions and emerged in relation to all themes. The themes are summarized in Table 1.

### 3.3. Cross-Cutting Themes

#### 3.3.1. Accessibility

Ideas Lab participants discussed substantial challenges to accessibility in healthcare, school, and other settings. These issues were exacerbated by stigma, discrimination, and provider ignorance about disability. Participants described challenges in accessing important information and essential services and supports, further contributing to their isolation. Existing services and resources were often hard to find, had bureaucratic barriers, lacked flexibility, or were not well coordinated. A practitioner participant indicated, “It’s complicated to fit everything together. For providers, it’s tough. For parents, it’s impossible”. Mothers described struggling to have their questions and concerns addressed and facing barriers such as service eligibility requirements or limited financial resources. As one mother explained, “There are so many barriers stacked on top of one another and it shouldn’t have to be so hard”.

Participants, particularly mothers, described a variety of ways in which services were hard to find and settings that were not readily accessible. Examples included birthing classes in which people with disabilities could not meaningfully participate, a lack of accessible equipment at doctors’ offices, and the unavailability of adaptive equipment (e.g., breast pumps). As one mother commented, “I felt very alone. Even in the birthing classes, I had to sit a lot of it out because I couldn’t participate, so I felt really alone”. Mothers frequently need to advocate for themselves and their children in health care settings. This can be challenging, as one mother who identifies as an advocate explained, “From the self-advocate side, I was at a doctor’s appointment yesterday and got treated horribly. You want to make sure when you go into anywhere, that you get properly introduced and aren’t discriminated against”. Many felt that professionals do not recognize mothers as experts on themselves and their children.

Recommendations regarding improvements in accessibility crossed themes related to treatment/services and systems, with implications for research. Participants described the need for providers to expect that people with IDD/BH will be parents and plan accordingly. Participants suggested that it would be helpful to provide more educational materials for parents with disabilities in accessible formats, for example, through videos, pamphlets, and visuals. In addition to the recommendation for interpreters for non-English speakers, providers and researchers must have greater knowledge and skill in interacting with parents with communication disorders. Participants advised that providers and researchers take the time to explain things better, in plain language, and break complex ideas into more manageable bullet points. Alternative solutions included having trained people to attend doctor appointments to advocate and interpret, having peer support in health care sessions, creating ombudsman positions within agencies, and incorporating community members as liaisons and engagement experts in research studies. Advocates could assist parents with service and systems navigation, helping to overcome administrative barriers. In terms of physical accessibility, participants suggested the benefits of accessible gathering places for activities such as playgroups. Individuals planning community events as well as those implementing research should routinely address challenges in accessibility.

#### 3.3.2. Diversity

One of the underlying unmet needs that emerged in workshop sessions was the lack of resources that highlighted the diversity of voices and experiences among mothers with IDD and behavioral health needs. Participants particularly focused on two key components of diversity as it pertained to improving treatment/services: first, better representation for Black, indigenous, and people of color (BIPOC) who are served in the disability sector, and second, the inclusion of disability as an identity under the diversity umbrella in all service sectors. A common thread throughout many conversations was that “nothing about us without us” should extend to both diversity and disability work, with one participant concisely stating, “I’m the key expert on me”. Ultimately, participants stressed that considering racial diversity as well as including disability in diversity efforts are critical goals for improving conditions for mothers with IDD and behavioral health needs.

Many participants noted the lack of diversity among the providers of services for mothers with IDD/BH needs, both in terms of race as well as disability. A common comment among participants was that many parents of color with disabilities feel even more discriminated against because of the lack of services that fit their specific cultural needs. Many participants brought up the power imbalance between them and their providers, and this was even more stark when there was no representation among providers. In the words of one mother, “If I’m at the table, does my voice matter?” Many parents said that this was a large barrier to creating trust with their providers, which made it difficult for them to reach their goals in care.

Not only did participants stress that the workforce was not racially diverse, but they also mentioned that it did not represent people with IDD/BH such as themselves. Many participants noted, ironically, the extreme difficulties they had in finding a job and how this was a major barrier to making the service workforce (or any workforce) more diverse for people with IDD/BH. One mother mentioned that it had taken over 100 job interviews to get a job and how difficult the process was, noting many of the administrative hurdles along the way. Others noted that, with these barriers in place, important roles such as peer support cannot be filled by people with lived experience. This is harmful to both the job seeker and the population of people seeking services.

Participants recommended that cultural competence and humility must be a focus of treatment/services and research. They suggested working closely with communities in need, possibly involving “cultural brokers” or people who are part of the community and understand its needs, language, culture, and dynamics, integrating color, culture, gender, disability, and behavioral health. These solutions will require deep, long-term investment in antiracism, along with knowledge of intergenerational trauma and its impact. Participants highlighted the importance of increasing the diversity of the workforce.

#### 3.3.3. Adverse Experiences and Trauma

Mothers and other participants described many examples of experiences that were challenging, negative, and, at times, traumatizing. They talked about feeling powerless, vulnerable, disrespected, blamed, and ignored, all of which contributed to feeling like “there is something wrong with you”. The potential for negative interactions with helping professionals and others who are meant to be supportive transcended settings, including health care, social services, schools, neighborhoods, and families. The emotional trauma resulting from these interactions undermined mothers’ feelings of wellbeing and confidence and, at times, kept them from reaching out again for health care, assistance, or support.

Participants explained that mothers are often vulnerable to stigmatizing language regarding their disabilities, along with the ageism and racism that may accompany health care experiences. As one participant described, “I see people with IDD are taken advantage of and I want to help”. Participants explained that providers often lack training and resources that would support mothers with IDD in their health care visits. Mothers often experience providers’ attitudes as negative, feeling that they are being judged and criticized. Consequently, participants described that mothers feel like they are always having to prove that they are right, particularly about the decisions they make.

Unfortunately, research is perceived as moving slowly and less focused on addressing these issues and having a real-world impact. Research participation itself can add to mothers’ trauma if not conducted in sensitive, respectful ways. Mothers explained that they are willing to participate in research if they perceive it as being helpful and if they are provided with choices regarding the ways in which they can participate. As one researcher explained, “In our research, we need to make sure parents aren’t overburdened”.

Participants made a number of recommendations for addressing and minimizing the impact of trauma. They suggested lessening stigma by talking with providers and others to increase awareness of the strengths and experiences of mothers with IDD/BH. Mothers encouraged other mothers to “recognize your values and gut instincts, and know and trust yourself”. Services should build on strengths, view the family as a whole, and empower mothers by advocating and offering choices. Mothers were encouraged to “surround yourself with people who are supportive” and identify natural supports as well as “unlikely” supporters and new partners.

Ground rules regarding language can be established at the beginning of meetings or health care visits, and are an essential consideration in research as well. Having an advocate accompanying the mother may reduce the likelihood of traumatization. Mothers underscored the importance of persistence: “Don’t be afraid to speak up; don’t be quiet; don’t give up; don’t back down”. Keeping a focus on longer-term goals can help mothers get through challenging or negative experiences in the short term.

#### 3.3.4. Trust

Throughout many of the workshop sessions, community experts stressed the fundamental importance of trust. Participants were asked to share about a time that they faced a challenge, whether as a mother, provider, policymaker, or researcher (or a combination of roles), and what would have helped them. Consistently, participants shared how trusting relationships helped them navigate challenges. This common sentiment was emphasized many times, along with the critical need for trust between parents and their providers. A mother explained, “If you find that people don’t trust their providers, then what? How does that amplify diverse voices?”

Many participants, mainly mothers or when speaking from the perspective of a mother, shared experiences that made them lose trust in providers and systems such as the health care system. They voiced that they were unable to advocate for themselves and that choices had been made on their behalf, which made them question the providers and systems that were supposed to be helping them. Trust was also frequently mentioned when discussing the concepts of diversity, disability, and working with diverse populations. Common themes included a lack of trust in communities, specifically between white communities and communities of color, as well as a lack of trust between patients of color and their (predominantly white) providers. Participants emphasized that trust is not “one size fits all” and that building trust does not happen overnight.

One practitioner participant noted that “when people feel trusted and validated, that relationship is paramount”. They mentioned that work, whether on an individual or community level, cannot happen without trust present and said that “without it, all other goals will not work”. Participants stressed that there are many different measures of success, including communication, but that trust is very important to them. Building trust within diverse communities was stressed as a major way to improve outcomes for mothers with IDD and behavioral health needs.

Participants recommended strategies for measuring trust from a research perspective. While there are instruments that provide for quantitative assessment, researchers and mothers agreed that there is a critical need for narrative storytelling along with quantitative data to acknowledge lived experiences. They mentioned that data collection on trust is best conducted in multiple ways or else the intervention or research project runs the risk of missing a significant part of the story on how trust is built and experienced. There was agreement that a finding such as “there is a lack of trust between patients and their providers” is important, but that the community should not be left alone with this finding. According to participants, researchers have an ethical and/or moral obligation to partner with the community to explore and address this lack of trust. One researcher emphasized, “If we are thinking about projects and resources, it can’t be what we think a community needs, but rather what the community needs at the center”.

### 3.4. Substantive Themes

#### 3.4.1. Services and Supports

Participants developed ideas for new interventions and programs for mothers with IDD/BH as well as those that could be based on existing models. Participants highlighted the benefit of concrete “hands-on” skills building. Interventions or services that mothers and children could access together were considered to have a potential positive impact for both. Participants recommended that services be available before crises occur (e.g., prevention programs). They suggested the value of peer support (i.e., either the enhancement of existing peer specialist models or the development of a new model specializing in IDD/BH). Participants recommended that providers be held accountable for having knowledge about and skills in working together with mothers with IDD/BH, and that potential payers be made aware and become involved in responding to the needs of mothers with IDD/BH. They suggested that mothers could receive services on a sliding fee scale. Language matters; support should be “normalizing”. Things should be explained better. Participants emphasized that mothers with IDD/BH conditions need choices. Participants’ specific recommendations for services and supports are provided in Table 2.

Participants agreed that mothers need to be involved in determining what is important to them. Participants suggested that outcome data include a mix of quantitative data, self-report measures, and personal stories/lived experiences. Potential prioritized outcomes included self-efficacy (mothers’ reports of “feeling adequate, confident” and their ability to articulate what they need); independence; and some measure of whether they are in crisis, as they wanted to reduce the number of crises they face. In addition, participants suggested that providers be encouraged to provide data on their practices, steps they are taking to improve practices, and whether practices are, in fact, improving.

#### 3.4.2. Peer Support

Participants described peer supports (e.g., peer specialists, peer advocates) as being critical for mothers with IDD/BH, and yet little support of this type, tailored to the perinatal period or parenting, was known to them. Participants highlighted the potential benefits of obtaining advice and support from other parents with experiences similar to theirs. Participants suggested that peer support programs could be staffed by mothers with IDD/BH who have already parented young children to help them with the challenges they faced. Peers could be helpful in advocating during meetings and navigating systems of care. One mother shared her challenge of obtaining a mental health diagnosis and discussed how peer support was essential to helping her through that period. Another mother discussed the challenges of interacting with the health care and other systems and stated that she “wished she had peer support to help her engage with those systems”. Participants described that they “want support…before they get into stressful situations”. As one participant relayed, “When all you do is parent—who takes care of you?” Despite this need, they were unaware of peer-to-peer support groups specific to parents with disabilities.

Participants agreed that a range of peer support (i.e., from informal social connections that reduce social isolation to professional peer specialist services) could be provided virtually and/or in person. Professional mentoring was also of interest to participants. Participants suggested that peer advocates who accompany mothers to health care or other types of appointments could help to prevent the “infantilization” of mothers with IDD/BH, as well as ensure that boundaries are respected by providers and other individuals with whom mothers come into contact. Peer support could also be made available to the families of mothers with IDD/BH to foster connections with other families.

Participants stressed that, in order for programs to be successful, “peer support needs to be valued monetarily”. Potential funders unfamiliar with peer support mechanisms may not value peer support and, therefore, may not be inclined to provide funding. It is also important to identify champions of peer support programs. Lastly, it may be challenging to identify, recruit, and hire individuals who could serve as peer supports/specialists. Individuals who provide peer support “need support, too”. Creating support to sustain the efforts of peer specialists was also perceived to be important.

Participants discussed building the evidence base of peer support program effectiveness. They suggested that researchers should focus on determining the number of parents served (or not being served), their needs, and how to reach and engage them; and that outcomes should include the health of their children; and the rate of removal of children from parents with IDD/BH by child welfare.

#### 3.4.3. Provider Practices and Training

The need for increased and improved training of health care and other service providers emerged as a key priority area among Ideas Lab participants. Participants described frequent challenges with what they characterized as providers’ lack of knowledge regarding disability across the lifespan, limited awareness of the needs of people with disabilities, limited ability to communicate in plain language or with people with communication disorders, and little familiarity with available resources. In addition, a few participants mentioned additional factors that may further complicate these dynamics, including the power differentials between health care providers and patients or clients, time and reimbursement constraints facing physicians, and the focus in medical education on a medical model of disability. As one participant explained, “The medical model doesn’t help providers work effectively with people with disabilities”. Participants emphasized the value of the social model of disability as an alternative model.

Participants discussed a variety of potential strategies to address these challenges, noting the importance of providing more training for providers overall. Specific suggestions included ensuring that such training is incorporated into medical education from the beginning, providing opportunities for providers to interact with parents with disabilities, and expanding existing training to include more disciplines and/or to focus on parents with disabilities as well as their children. Participants emphasized the importance of building on existing resources and connecting with groups already doing this work, including medical and nursing schools and associations, advocacy groups, and others.

Ideas Lab participants envisioned these strategies as ways to move toward a world in which health care and other systems are accessible and parents with disabilities feel seen, partnered with, and able to have their needs met. They also envisioned that providers would develop a more positive view of people with disabilities and their ability to parent, understand what supports they might need, and know what resources are available.

Participants suggested creating patient/client satisfaction surveys and provider questionnaires to help monitor progress toward these goals, provide accountability, and determine where additional support is needed. They indicated that research could help by developing these surveys and self-report measures, documenting the effectiveness of different programs and interventions, and helping to determine what is missing in interactions between patients/clients and providers. They further recommended the establishment of communities of practice across systems and states to share models, support practice innovation, and build the evidence base.

#### 3.4.4. Systems Navigation and Transformation

Participants discussed challenges in navigating service systems and implementing systems change, with too few supports available for mothers with IDD/BH to help with navigating health care and other systems. Participants explained that people do not know where to go for help. The lack of coordination across sectors and systems, and the complexities within systems and agencies, make integrated care planning and service access difficult and often serve as barriers to successful engagement and potential benefits, according to participants. Eligibility requirements and income limits were among the bureaucratic barriers cited. Parents with IDD/BH, often the “end users,” are not fully engaged as partners in the planning, organization, or implementation of services. Participants recommended that systems and service sectors address what might be considered health-related social needs that serve as barriers to care, including access to transportation and affordable childcare and challenging Social Security income limits.

Participants recommended that preventive approaches would be preferred to crisis approaches, as “situations aren’t addressed until it is almost too late”. Parenting services are often available in the child welfare system, but only when you are perceived to have “failed” as a parent (i.e., by being suspected of neglecting or abusing your child). Unfortunately, participants described experiences of being caught up in the child welfare system because of the stigma, negative attitudes, and assumptions associated with disability and behavioral health conditions. Participants described a disconnect between parents’ priorities and needs, treatment and research goals, and prioritized outcomes. Participants’ recommendations for systems navigation and transformation are provided in Table 3.

### 3.5. Research Recommendations

Research recommendations were generated in all discussions and cut across all themes, as described above. Many participants underscored the lack of data on many of the topics they were interested in, such as the number of parents receiving treatment or served through various programs, the demographic characteristics of parents receiving various services, and evidence to support the effectiveness of interventions, services, and supports. Additionally, many participants emphasized the need for a comprehensive needs assessment for mothers with IDD/BH, centering the needs of mothers and their families to fully understand the diversity, breadth, and depth of needs of mothers in this community.

Participants acknowledged that an important step when addressing the experiences and needs of mothers with IDD/BH is working together with researchers to incorporate stakeholders’ voices into the research agenda. Many researcher participants expressed concern that researchers, in general, lack the skills necessary to incorporate mothers’ voices into their work fully. Importantly, participants recommended that researchers’ skills be strengthened to support engagement with the target population of mothers with IDD/BH and community experts. Participants’ experiences suggested that researchers may require guidance and coaching to build their competencies in community-engaged work (e.g., to present their ideas and requests in clear and understandable ways).

These comments underscore the importance of expanding not only the inclusion of mother-driven questions and priorities in the research agenda but also of strengthening researcher training and skills to engage mothers with IDD/BH and other community members actively, in meaningful ways. Many researchers discussed the general lack of trust, both in research and the medical field, and how additional training for researchers could help address some of these important concerns and overcome barriers to mothers’ research participation. Research recommendations are summarized in Table 4.

## 4. Discussion

Ideas Lab participants corroborated themes identified in prior literature related to the experiences and vulnerabilities of mothers with IDD, and challenges in accessing and engaging in treatment [14,20,21]. They, indeed, described a lack of treatment and services that acknowledge their strengths or are tailored to their needs. Co-occurring behavioral health conditions add an additional layer of stigma which, in participants’ experiences, may fuel the negative attitudes of providers and serve as a further barrier to mothers’ help-seeking. Ideas Lab participants provided significant detail regarding the ways in which cross-cutting issues of accessibility, diversity, adverse experiences and trauma, and trust contribute to or undermine their engagement with services, supports, and research. Cultural competence and humility were emphasized.

Issues of identity figured significantly in Ideas Lab discussions. The lack of attention to diversity in race, gender, culture, or spoken language, for example, of mothers with IDD/BH or of service providers themselves, undermined the accessibility and trust essential to treatment access and engagement, and potentially contributed to adverse experiences in treatment settings. Mothers with IDD/BH identified as “more” than simply their disabilities and “more” than individuals, with conversations about family needs predominating. While the initial focus of Ideas Lab discussions was on maternal health and wellbeing, conversations quickly shifted to the experiences and needs of children and families, underscoring the impact of family wellbeing on the health and wellbeing of women as mothers and the need for family-focused treatment approaches. Participants agreed that mothers must be involved in determining what is important in terms of outcomes, both for themselves and for their children.

Ideas Lab participants generated a list of recommendations for specialized and tailored treatment and services, focusing on the perinatal period, parenting, and more generic issues of self-care and relationships with partners. Their experiences with and recommendations for advocacy skills and resources were applicable across the lifespan and in diverse settings. The potential benefit of peer support and mentoring were highlighted, again confirming the notion, suggested by others (e.g., autistic-delivered peer support for employment), that peer support holds promise for future development and testing [29].

Participants all agreed on the need for increased and enhanced training for service providers, which early intervention providers themselves have recently identified as a gap [30]. Ideas Lab participants acknowledged that providers often work in contexts that complicate their efforts. Health care providers may be constrained by time and reimbursement requirements, for example. The social model of disability was offered as an alternative to the medical model and suggested as a way of offering providers a more positive approach to mothers with IDD/BH. Measures and methods were recommended for monitoring changes and progress in training and practice.

The lack of coordination across service sectors and systems contributes to accessibility and navigation issues, as reported by Ideas Lab participants. Again, participants recommended a “whole family” approach, wrapping services around families and addressing health-related social needs. Participants highlighted the importance of identifying champions to help make policy changes and engaging with community leaders and legislators to inform their efforts.

Research recommendations cut across all themes and domains of interest. Participants highlighted the lack of data regarding prevalence, needs, and the effectiveness of treatment and services for this vulnerable population. Ideas Lab participants were explicit in suggesting that research methods and measures be accessible and sensitive to the needs and preferences of mothers with IDD/BH, and that outcomes be relevant and meaningful to them. Recommendations included ways in which mothers with IDD/BH and other community experts can be engaged in crafting research methods and measures as well as in implementing and disseminating research findings. For example, mothers with IDD/BH can be engaged in developing and evaluating training programs for providers.

The project had a number of limitations. The Ideas Lab group was relatively small (*n* = 30), although group members met for a total of 10 h, with numerous planning and one-on-one discussions with participants between sessions. The Ideas Lab sessions were conducted virtually, which allowed for geographic diversity, though may have impacted discussions in some way. The virtual format, however, supported a range of accommodations for communication preferences (e.g., closed captioning, text entries in the chat, recording for potential replaying of the sessions) and supported the engagement of participants who may have otherwise had childcare or transportation needs. While Ideas Lab participants overwhelmingly identified as women and mothers who had given birth, project findings and recommendations must be further considered as they relate to men and in the context of the experiences of trans, cis-gender, and non-binary people with IDD/BH who identify as parents who may or may not have given birth, with tailored services and supports developed and tested to build an evidence base sensitive to the diversity of parents and disabilities. In addition, attention should be paid to the experiences of mothers of diverse races, ethnicities, languages, and cultural backgrounds, particularly given the likely disparities in treatment access and service utilization undermining positive outcomes for these mothers and their children.

The Ideas Lab Implementation Team, working in partnership with KI facilitators, was a strength, contributing both experience and expertise in anticipating and meeting the needs of mothers with IDD/BH and community experts, particularly regarding communication preferences and engagement. The Implementation Team and many Ideas Lab participants continue to be engaged in the work of the MMHRC, highlighting their concern for the experiences and needs of mothers with IDD/BH, their commitment to addressing gaps in treatment and services, their recognition that all stakeholders are community experts who have experience and expertise to offer providers, and their enthusiasm for engaging with researchers in partnership efforts throughout the research life cycle.

## 5. Conclusions

While mothers with intellectual and developmental disabilities and behavioral health conditions may be considered a vulnerable population, they are interested in sharing their experiences and expertise and “having a voice”. Mothers with IDD/BH participated in five virtual Ideas Lab workshop sessions with additional community experts, including family members, advocates, providers, policymakers, and researchers, to discuss their needs and those of their families. Ideas Lab participants highlighted the importance of attending to issues of accessibility, diversity, adverse experiences and trauma, and trust in service provision, systems navigation, and research. Stakeholders, who reflected multiple perspectives (i.e., mothers, providers, and researchers were not mutually exclusive groups), underscored the importance of identity, cultural sensitivity and humility, and a family-focused approach in efforts to improve inter-dependent health and wellbeing outcomes for mothers and their children. All participants contributed to recommendations for the development, implementation, and study of innovative treatment and services, including peer support, improved provider training, and facilitated systems navigation and transformation. Ideas Lab participants recommended that mothers with IDD/BH be included from the beginning of research and development efforts to ensure the relevance and enhance the ultimate impact of treatment innovation, policy development, and research findings.

## Figures and Tables

**Table 1:** Ideas Lab participants’ emerging themes.

Cross-Cutting Themes	Substantive Themes
Accessibility: Challenges in accessing important information and essential services and supports	Services and Supports: New/adapted interventions and programs for mothers and children
Diversity: Lack of attention and support at the inter-section of race and disability	Peer Support: Support from those with similar experiences, particularly in the perinatal period
Adverse Experiences and Trauma: Negative experiences and interactions resulting in trauma, undermining wellbeing and confidence	Provider Practices and Training: Increased and improved training of health care and other service providers
Trust: Lack of trust in communities, providers, and relationships	Systems Navigation/Transformation: Improved coordination across and within complex sectors and systems

**Table 2:** Ideas Lab participants’ recommendations for treatment and services.

Treatment/Services Recommendations	Considerations for Mothers with IDD/BH
Specialized doula services	Doulas with advanced skills and understanding of mothers with IDD/BH could be employed in existing programs/services or new specialized programs could be developed and paid for by Medicaid or other insurers. Birth plans were suggested as having a potential benefit to mothers, with a positive impact on babies. A further suggestion was that mothers with IDD/BH may benefit from having a support person or advocate to attend perinatal/doctor’s appointments with them.
2. Tailored perinatal psychiatric consultation	Models to provide psychiatric consultation in the perinatal period could be adapted or enhanced to provide consultations specific to mothers with IDD/BH. This consultation service should be paid for by insurers.
3. Accessible birthing classes	Classes tailored to the physical, intellectual, and emotional strengths and challenges of mothers with IDD/BH would support meaningful participation and contribute to improved outcomes for mothers and babies.
4. Home visiting programs	Existing home visiting programs (e.g., nurse–family partnership home visitation programs) could be adapted or enhanced for mothers with IDD/BH, with the potential for support from Medicaid or other payers (e.g., state agencies). Visiting nurses with expertise in IDD/BH conditions could be associated with physicians managing the pregnancies of mothers with IDD/BH, often considered “high risk”.
5. Self-care and healthy relationships	Participants recommended programs or interventions to support mothers themselves, including a focus on self-care and developing and sustaining healthy relationships (e.g., dating, with partners, with family members and children). They emphasized the importance of focusing on the “person,” rather than the “disability”. Self-care interventions should include information, education, and concrete action steps.
6. Advocacy skills and resources	Mothers must understand how to advocate for themselves and their children, within the family as well as with agencies and service providers. Mothers recommended a focus on concrete skills. A template for questions to have in hand before going to an appointment would be helpful.

**Table 3:** Ideas Lab participants’ recommendations for systems navigation and transformation.

Systems Navigation/ Transformation Recommendations	Considerations for Mothers with IDD/BH
Wrap services around families	Provide “one-stop shopping” for services and support for parents, children, and families. Encourage person-centered planning and supported decision-making, acknowledging that parents are the experts on their children. Lower bureaucratic barriers to accessing services. Establish multisystem and cross-sector partnerships with providers, school systems, parents, and advocacy organizations (e.g., learning collaboratives, communities of practice). Secure funding support from state agencies and Medicaid.
2. Address health-related social needs	For example, improve public transportation, bus shelters, and accessible transportation options. Work with state agencies to improve education outcomes and employment rates for parents. Increase funding for IDD and IDD-related efforts.
3. Increase awareness and spread knowledge	Inform the agendas of agencies, non-profits, and parent groups regarding the experiences, needs, and capabilities of mothers with IDD/BH and their families. Create opportunities for parents, families, individuals, and allies to provide input and feedback when creating new policies and programs.
4. Create and mobilize networks of resources and supports	Invite additional and non-traditional stakeholders to participate in reflecting on and integrating the experiences, needs, and capabilities of mothers with IDD/BH and their families. Identify champions, leaders, key informants, and content experts and engage them in this work.
5. Initiate and support legislative action	Inform legislative representatives to “get them on board” with the issues facing parents with IDD/BH and what they need to succeed. Connect with government officials and legislative commissions. Promote legislation to ensure that parents do not lose custody of their children simply because they live with disabilities. Meet with candidates running for office. Develop a simple platform and clear message regarding the experiences and needs of mothers with IDD/BH.

**Table 4:** Ideas Lab participants’ recommendations for research.

Ideas Lab Themes	Research Recommendations
Accessibility	Consider mothers’ communication styles and preferences, particularly as they are engaged and involved in research. Develop relevant, meaningful, valid, and reliable ways to measure factors that matter to mothers with IDD/BH. Identify and test ways to make existing services and supports more accessible.
Diversity	Compile demographic data on mothers with IDD/BH and their families receiving services or with unmet needs. Explore the background, characteristics, and culture of parents and the barriers they face.
Adverse Experiences/ Trauma	Recognize and respect that stakeholders (including researchers) have multiple roles and experiences that may not fit neatly into categories. Conduct research in trauma-informed ways, taking care to provide accessible information about purposes, methods, procedures, and potential impacts.
Trust	Consider the “end user” as a partner in the development and implementation of research. Create a validated measure of trust, relevant to members of diverse communities. Include trust as a measure of success in research. Study trust in the context of provider–mother relationships and interactions.
Services and Supports	Develop or adapt and test intervention models (e.g., doulas, perinatal psychiatric consultations, advocacy skills, self-care and healthy relationships, disability/psychoeducation for children and family members). Employ a mix of quantitative and qualitative data, including mothers’ stories and self-report measures. Identify and target outcomes that matter to mothers with IDD/BH (e.g., increased self-efficacy, decreased number and extent of crises). Include outcomes such as number of parents served, data on who/where/how parents are reached, health of children, and child (child welfare) removal rates.
Peer Support	Conduct needs assessments addressing questions such as the following: Who is the ideal peer? What do parents need from peer support? How can it help? Where should it take place? How can parents and potential peer support specialists be reached? Conduct a scoping review of existing peer support programs that can be learned from or built on, as well as available networks of peer support or mentors. Determine what works and what does not work, from mothers’ perspectives. How can existing models be tailored to mothers with IDD/BH and their families? Build an evidence base of peer support program effectiveness by collecting data on parents served and relevant outcomes (e.g., including health of children, child removal rates).
Provider Practices and Training	Investigate what is missing or what could be improved in interactions between mothers and providers. Survey healthcare providers about their needs. Survey provider associations to determine the prevalence of mothers with IDD/BH, those served, and those who would benefit from additional, tailored services. Study providers’ practices, the steps they are taking to improve practices, and whether practices are improving. Engage mothers in developing training programs and evaluating their effectiveness. Administer satisfaction surveys after appointments.
Systems Navigation/ Transformation	Explore the needs (met and unmet) of mothers with IDD/BH and their families. Study service utilization by mothers with IDD/BH. Explore ways to overcome barriers to service access and use. Explore factors incentivizing and supporting cross-system or cross-sector collaboration and service integration.

## Data Availability

Data are unavailable.
